# Supplementary material for: Sample collection and transport strategies to enhance yield, accessibility, and biosafety of COVID-19 RT-PCR testing
Source: J Med Microbiol. 2021 Sep 6;70(9):001380. doi: 10.1099/jmm.0.001380 (PMC8697510; doi:10.1099/jmm.0.001380)
Supplement: Supplementary material 1 [file jmm-70-1380-s001.pdf]

	Individuals with all negative samples (N=13)
**Mean Age in years (SD)**	57 (15)
**# of Men (%)**<br>**# of Women (%)**	8 (61%)<br>5 (39%)
**Ethnicity (%)**<br>Hispanic<br>Black<br>White	 5 (39%)<br>6 (46%)<br>2 (15%)
**Comorbidities**<br>Hypertension<br>Diabetes Mellitus<br>Coronary Artery Disease<br>Chronic Kidney Disease<br>Lung Disease (eg, COPD)<br><br>No chronic disease	 5 (39%)<br>2 (15%)<br>1 (8%)<br>3 (23%)<br>2 (15%)<br><br>5 (38%)
**COVID symptoms (%)**<br>Cough<br>Shortness of breath<br>Fever<br>Diarrhea<br>Chest Pain<br><br>No COVID symptoms	 3 (23%)<br>4 (31%)<br>4 (31%)<br>2 (15%)<br>0 (0%)<br><br>8 (62%)
**Oxygen Support Required (%)**<br>None<br>Nasal Canula<br>Mechanical Ventilation	 9 (69%)<br>0 (0%)<br>4 (31%)<br>0 (0%)
Symptom duration prior to baseline collection<br>**Mean (range)**	 12 days<br>(4 – 28 days)
Days between in-hospital NP swab PCR and baseline collection: **mean (range)**	3 days<br>(1 – 5 days)
Number of follow-up time-points per participant: **mean (range)**	0
Participants with negative NP swab PCR collected in clinical follow-up during hospitalization	6 (50%)

Table S1. Characteristics of participants with all negative samples.

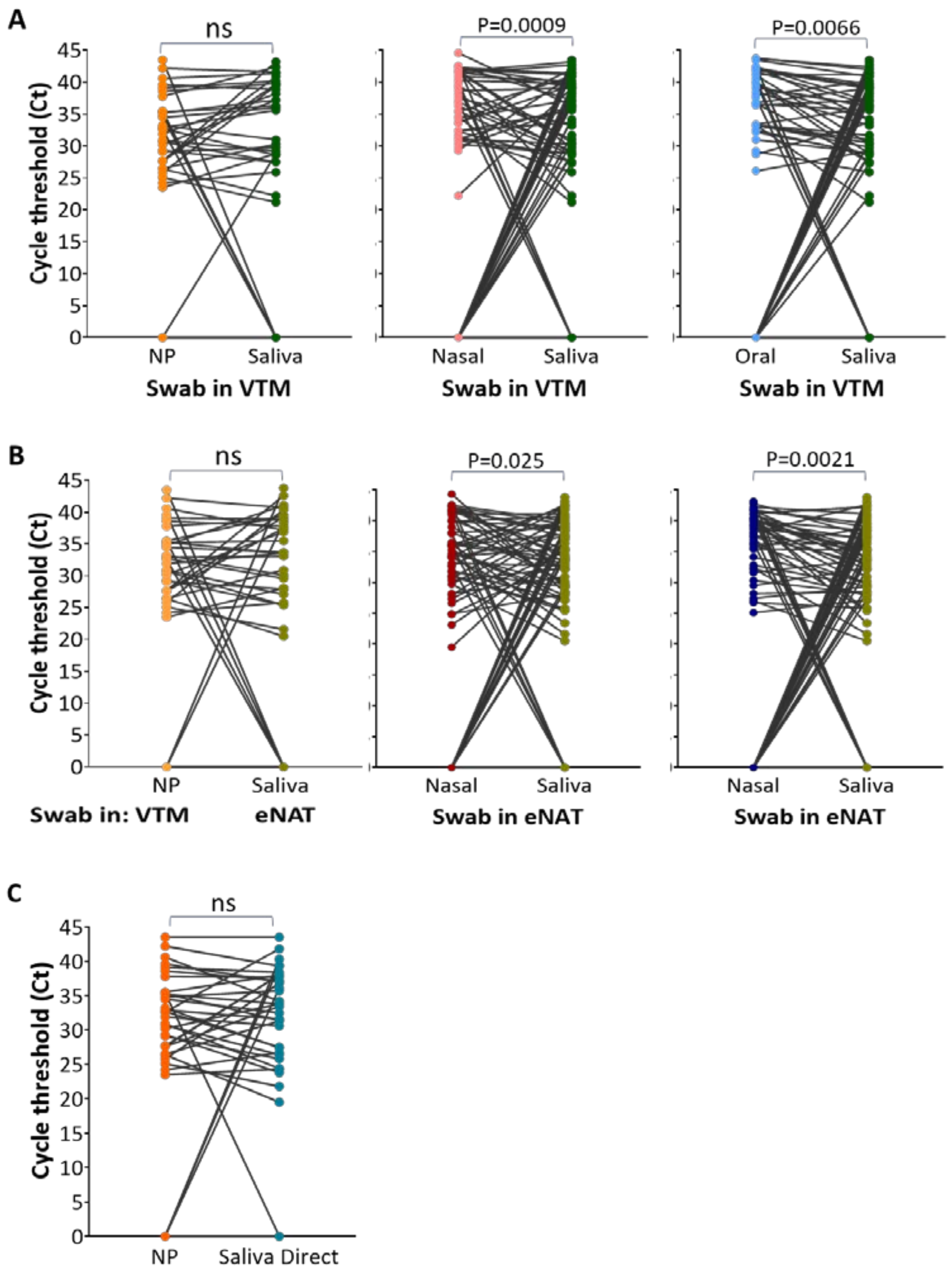

Fig. S2. Paired sample sets showing Ct values comparing each respiratory sample collected in (A) VTM, N=84 and (B) eNAT, N=84. (C) Comparing nasopharyngeal specimens with direct saliva (N=37). The lines indicate samples from the same patient collected at the same time point. ns=not significant.

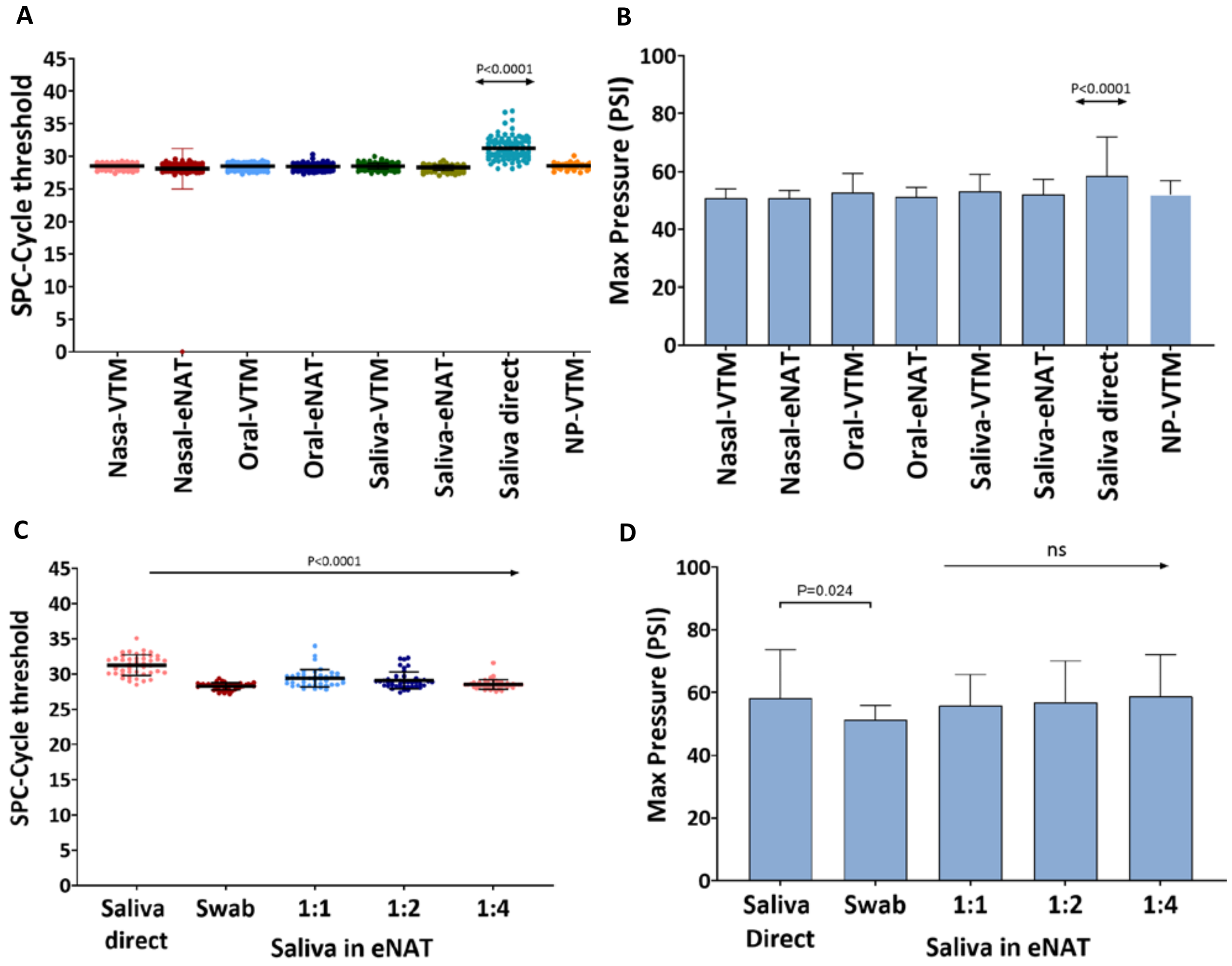

Fig. S3. (A and C) Internal control (SPC) cycle threshold (Ct) values for all respiratory specimens (A, N=84) and for selected saliva samples diluted in eNAT (C, N=44); (B and D) In-cartridge maximum pressure values (PSI) for different respiratory specimens (B, N=84) and for selected saliva samples diluted in eNAT (D, N=44). Each sample tested is represented by points (♦) on the scatter plot and the lines show the mean and standard deviation values. ns= not statistically significant.
